# Differences in Left Ventricular Global Function and Mechanics in Paralympic Athletes with Cervical and Thoracic Spinal Cord Injuries

**DOI:** 10.3389/fphys.2016.00110

**Published:** 2016-03-29

**Authors:** Katharine D. Currie, Christopher R. West, Andrei V. Krassioukov

**Affiliations:** ^1^Autonomic Research Laboratory, International Collaboration on Repair Discoveries, Faculty of Medicine, University of British ColumbiaVancouver, BC, Canada; ^2^Translational Integrative Physiology Laboratory, Faculty of Education, School of Kinesiology, University of British ColumbiaVancouver, BC, Canada; ^3^Division of Physical Medicine and Rehabilitation, Faculty of Medicine, University of British ColumbiaVancouver, BC, Canada; ^4^GF Strong Rehabilitation Centre, Vancouver Coastal HealthVancouver, BC, Canada

**Keywords:** athletes, echocardiography, paraplegia, strain, stroke volume, tetraplegia

## Abstract

Following a spinal cord injury, there are changes in resting stroke volume (SV) and its response to exercise. The purpose of the following study was to characterize resting left ventricular structure, function, and mechanics in Paralympic athletes with tetraplegia (TETRA) and paraplegia (PARA) in an attempt to understand whether the alterations in SV are attributable to inherent dysfunction in the left ventricle. This retrospective study compared Paralympic athletes with a traumatic, chronic (>1 year post-injury), motor-complete spinal cord injury (American Spinal Injury Association Impairment Scale A-B). Eight male TETRA wheelchair rugby players (34 ± 5 years, C5-C7) and eight male PARA alpine skiers (35 ± 5 years, T4-L3) were included in the study. Echocardiography was performed in the left lateral decubitus position and indices of left ventricular structure, global diastolic and systolic function, and mechanics were derived from the average across three cardiac cycles. Blood pressure was measured in the supine and seated positions. All results are presented as TETRA vs. PARA. There was no difference in left ventricular dimensions between TETRA and PARA. Additionally, indices of global diastolic function were similar between groups including isovolumetric relaxation time, early (E) and late (A) transmitral filling velocities and their ratio (E/A). While ejection fraction was similar between TETRA and PARA (59 ± 4 % vs. 61 ± 7 %, *p* = 0.394), there was evidence of reduced global systolic function in TETRA including lower SV (62 ± 9 ml vs. 71 ± 6 ml, *p* = 0.016) and cardiac output (3.5 ± 0.6 L/min vs. 5.0 ± 0.9 L/min, *p* = 0.002). Despite this observation, several indices of systolic and diastolic mechanics were maintained in TETRA but attenuted in PARA including circumferential strain at the level of the papillary muscle (−23 ± 4% vs. −15 ± 6%, *p* = 0.010) and apex (−36 ± 10% vs. −23 ± 5%, *p* = 0.010) and their corresponding diastolic strain rates (papillary: 1.90 ± 0.63 s^−1^ vs. 1.20 ± 0.51 s^−1^, *p* = 0.028; apex: 3.03 ± 0.71 s^−1^ vs. 1.99 ± 0.69 s^−1^, *p* = 0.009). All blood pressures were lower in TETRA. The absence of an association between reduced global systolic function and mechanical dysfunction in either TETRA or PARA suggests any reductions in SV are likely attributed to impaired loading rather than inherent left ventricular dysfunction.

## Introduction

The classification of Paralympic athletes is sports specific and considers physical, visual and/or intellectual impairments. Typically athletes with a spinal cord injury (SCI) are competing against individuals with a similar lesion level (i.e., cervical or thoracic SCI). However, there are sporting competitions where individuals with cervical and thoracic SCI are competing against each other, in addition to other non-SCI athletes. The consideration of how the lesion level may influence exercise performance is therefore necessary to ensuring fair competition between the diverse population of Paralympic athletes.

One contributing factor to exercise performance is the capacity of the cardiovascular system to adequately respond to the body's demands. This includes the responses of the heart, vasculature, and adrenal medulla. Following SCI, there are lesion level dependent alterations in how the cardiovascular system responds to an exercise stimulus. While sympathetic preganglionic neurons originate in the thoracic and upper lumbar segments of the spinal cord (T1-L2), sympathetic innervation of target organs is segmental. Innervation of the heart and vascular smooth muscle of the upper body arise from the high thoracic segments (T1-T5), while innervation of the vascular smooth muscle of the lower body and adrenal medulla arise from the lower thoracic and lumbar segments (T6-L2). Cervical SCI results in the most dramatic alterations in cardiovascular outcomes at rest and in response to exercise, due to the disruption of descending sympathetic input to target organs below the level of injury (Krassioukov, 2009). We recently confirmed this by demonstrating individuals with tetraplegia who had an autonomic complete SCI also reached attenuated peak heart rates of 102 ± 34 bpm while individuals with tetraplegia and autonomic incomplete SCI reached higher peak heart rates (161 ± 20 bpm) (Currie et al., 2015). Additional cardiovascular responses that are attenuated during exercise in individuals with tetraplegia include the changes in blood pressure (Thijssen et al., 2009), catecholamine levels (Schmid et al., 1998a,b), and stroke volume (SV) (Kessler et al., 1986; Hostettler et al., 2012). Contrary to tetraplegia, individuals with high and low paraplegia have either partial or full preservation of descending sympathetic pathways to the heart, vascular smooth muscle and adrenal medulla, and therefore are capable of mounting an appropriate cardiovascular response during exercise. Previous research has demonstrated individuals with paraplegia are capable of reaching peak heart rates in range of their age-predicted heart rate maximum (Hopman et al., 1992, 1993; Jacobs et al., 2002) and display exercise-induced increases in circulating catecholamines (Schmid et al., 1998a,b) and blood pressure (Dela et al., 2003; Claydon et al., 2006). SV responses to aerobic exercise in individuals with paraplegia, however, are inconclusive with previous research either demonstrating no change (Davis and Shephard, 1988; Hopman et al., 1993; Raymond et al., 2001; Theisen et al., 2001) or small increases (Davis et al., 1987; Hopman et al., 1992; Raymond et al., 1999). Overall, exercise-induced increases in cardiac output ($\dot{Q}$) in individuals with SCI are primarily driven by changes in heart rate, with individuals with paraplegia exhibiting a greater capacity to increase $\dot{Q}$ during exercise.

Altered SV responses in SCI have primarily been attributed to a reduction in loading (i.e., decreased venous return) (Theisen, 2012). However, no studies have examined whether factors inherent to the left ventricle (LV) *per se* are also a contributing factor. Echocardiographic assessments of LV mechanics are a non-invasive tool that may help to determine if changes in LV performance following SCI are associated with SV reductions. Indices of LV mechanics include strain (ε) and strain rates (SR) which assess the degree and rate of myocardial deformation in longitudinal, radial and circumferential axes; rotation (Rot) and rotation rates (RotR) of the base and apex of the LV; and overall LV twist and twisting/untwisting rates (Voigt et al., 2015). In clinical conditions, such as chronic heart failure, there is a concomitant reduction in global LV systolic function [SV; LV ejection fraction (EF)] and LV systolic mechanics (Leung and Ng, 2010; Ma et al., 2014), and recent evidence suggests lower ε in these patients is associated with a reduced exercise capacity (Hasselberg et al., 2015). Additionally, indices of LV mechanics are sensitive to increases and decreases in sympathetic activity (Weidemann et al., 2002; Akagawa et al., 2007), which may provide insight into the influence of lesion level on LV performance. Few studies have performed echocardiographic assessments in athletes with SCI (Huonker et al., 1998; Gates et al., 2002; Schumacher et al., 2009; Maggioni et al., 2012; West et al., 2012a; De Rossi et al., 2014), with none of these investigations examining LV mechanics. Given evidence that global LV systolic dysfunction is associated with impairments in LV mechanics, and that these functional changes may limit exercise performance, the examination of LV structure, function and mechanics in athletes with SCI may help to elucidate potential factors limiting cardiac responses to exercise. Therefore, the purpose of this study was to compare resting LV structure, function and mechanics in high-performance athletes with SCI.

## Materials and methods

### Participants

This was a retrospective comparison of echocardiography data collected at both summer and winter Paralympic events. Inclusion criteria included males between 18-60 years of age who had sustained a chronic (>1 year post-injury), motor-complete [American Spinal Injury Association Impairment Scale (AIS) A-B], traumatic SCI, and who have been competing at the international level for at least 3 years. Eight Paralympic wheelchair rugby players with a cervical SCI (TETRA) and eight Paralympic alpine skiers with a thoracic SCI (PARA) met the criteria and were included in the study. The neurological level of injury and AIS was confirmed using the International Standards for Neurological Classification of SCI (Kirshblum et al., 2011). Exclusion criteria for the study included any history of cardiovascular disease or acute illness/infection, which was confirmed with a verbal medical history, and any language or cognitive barrier that prevented the participant from following English instructions. The International Paralympics Committee, University of British Columbia Clinical Research Ethics Board, and Brunel University Research Ethics Board approved all protocols, which conformed to the Declaration of Helsinki, and all individuals provided written informed consent prior to participation. Prior to testing, participants were instructed to abstain from food and drink for 4 h, alcohol and caffeine for 12 h, and exercise for 24 h. On the day of testing, all participants were instructed to void their bladder to reduce the potential influence of sympathetic reflex activation on blood pressure.

### Blood pressure assessments

Blood pressures were measured from the left brachial artery using an automated machine (Dinamap Pro 300 V2; GE Healthcare, Milwaukee, USA). Seated and supine measurements were collected in duplicate following 5 and 10 min of quiet rest, respectively.

### LV assessments

Cardiac images were collected with the participants in the left lateral decubitus position using two-dimensional echocardiography (Vivid 7; GE Healthcare, Horton, Norway) according to the recommendations of the American Society for Echocardiography (Quiñones et al., 2002; Lang et al., 2005; Voigt et al., 2015). Heart rate was measured continuously using a single-lead electrocardiogram. Images were analyzed offline using dedicated software (EchoPAC; GE Healthcare, Horten, Norway) by a single blinded investigator, and the average of three cardiac cycles is presented.

Indices of LV structure were measured at end-diastole (d) and end-systole (s) from the parasternal long axis view, including LV internal diameter (LVID), and interventricular septal wall (SWT), and posterior wall (PWT) thickness. Relative wall thickness (RWT) was calculated as [(2 × PWTd)/LVIDd]. LV mass (LVMI) was calculated according to an established formula (Devereux et al., 1986), and indexed to body surface area (Du Bois and Du Bois, 1989). Modified single-plane Simpson's method was used to analyze apical four-chamber views to determine end-diastolic (EDV) and end-systolic (ESV) volumes, and the global systolic function outcomes of SV, EF, and $\dot{Q}$ which was calculated as the product of SV and heart rate. Global diastolic function outcomes were determined from pulsed-wave Doppler at the tips of the mitral valve leaflet. Outcomes included early (E) and late (A) transmitral filling velocities and their ratio (E/A), and isovolumetric relaxation time (IVRT).

Indices of LV mechanics were derived from 2D speckle-tracking analysis of apical four-chamber and parasternal short axis images at the level of the mitral valve (basal), papillary muscle (mid), and apex (apical) using established guidelines (Mor-Avi et al., 2011). Using a cubic spline algorithm, raw speckle-tracking traces were interpolated by customized post-processing software (2D Strain Analysis Tool, Stuttgart, Germany) into 600 points in systole and 600 points in diastole to control for heart rate differences. Peak ε and SR in systole and diastole were determined from short-axis (radial, circumferential) and four-chamber (longitudinal) images. Peak Rot and RotR in systole and diastole were determine at the basal and apical levels, and twist was determined as the maximum value from subtracting frame-by-frame basal and apical Rot data. Peak systolic twisting velocity and early diastolic untwisting velocity were determined the same way using frame-by-frame basal and apical RotR data.

### Statistical analyses

Statistical analyses were performed using Statistical Package for Social Science software (IBM Corporation, Armonk, NY, USA). Data were assessed for normality using Shapiro-Wilk tests and Q-Q plot analyses. Between group differences were determined using independent *t*-tests and Mann-Whitney *U*-tests for normally and non-normally distributed data, respectively. Effect sizes were calculated using Cohen's d. Data are presented as mean ± SD unless otherwise noted, with *P* < 0.05 considered statistically significant.

## Results

**Figure 1:**
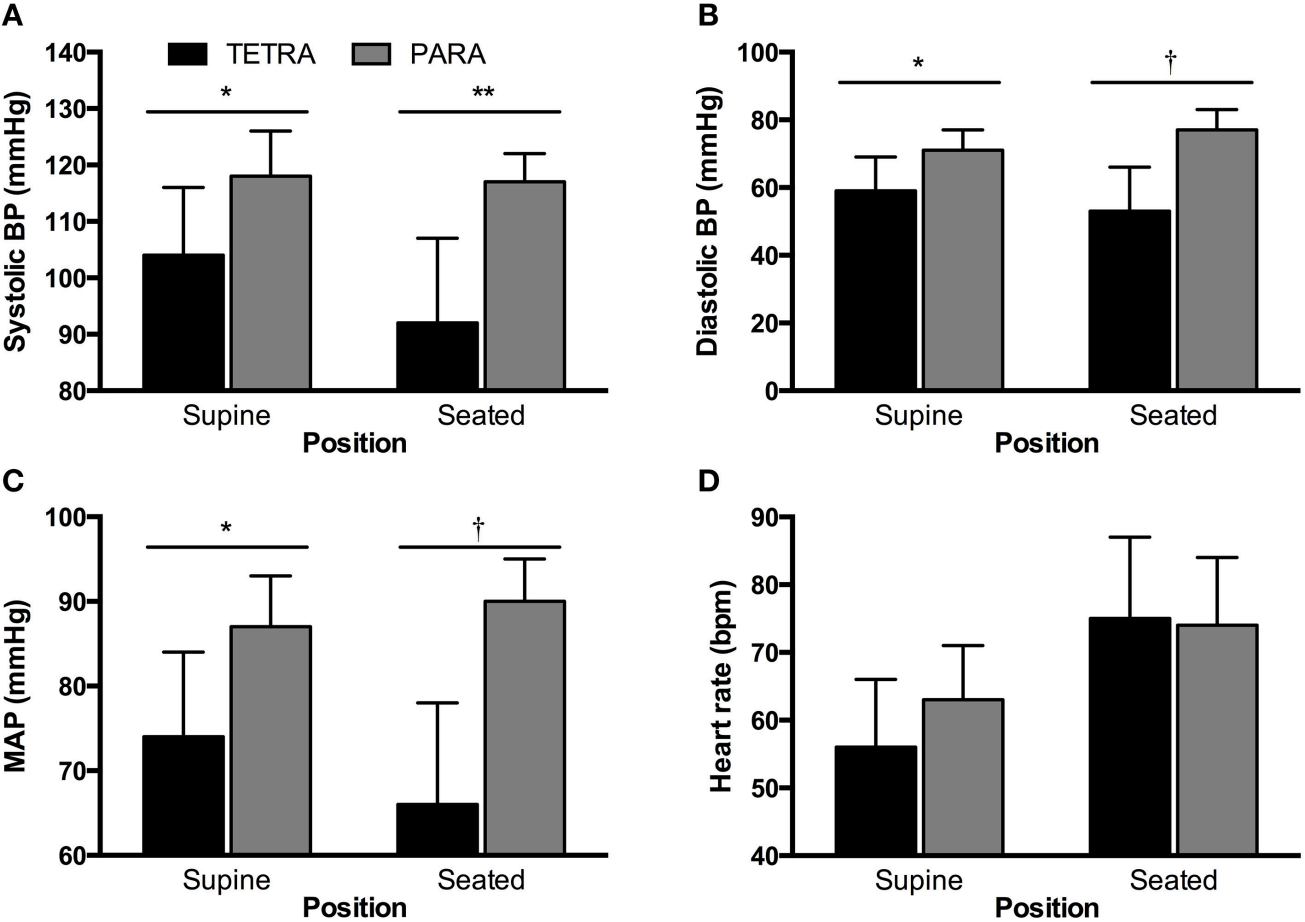
**Supine and seated systolic (A) and diastolic (B) blood pressure (BP), mean arterial pressure (C, MAP) and heart rate (D) for TETRA (black bars) and PARA (gray bars) groups**. Between-group differences: ^*^p < 0.05; ^**^p < 0.01; †*p* ≤ 0.001.

Individual and group values for participant characteristics are presented in Table 1. Body surface area was similar in TETRA (1.84 ± 0.13 m^2^) and PARA (1.93 ± 0.15 m^2^, *p* = 0.232). Self-reported training history was available in 5 of 8 TETRA and all 8 PARA athletes. There was no significant difference in the number of years competing at an international level (TETRA: 11 ± 4 years; PARA: 8 ± 5 years, *p* = 0.275) or weekly training volume (TETRA: 10 ± 3 hours/week; PARA: 16 ± 13 hours/week, *p* = 0.192). For seated and supine hemodynamics, heart rates were similar between groups while all blood pressure values were lower in TETRA compared to PARA (Figure 1).

**Table 1:** **Participant characteristics**.

	**Lesion Level**	**AIS**	**TPI (yr)**	**Age (yr)**	**Height (m)**	**Mass (kg)**
**TETRA**						
1	C5	A	14	38	1.68	61
2	C6	A	11	38	1.76	62
3	C6	A	12	37	1.76	63
4	C6	B	12	25	1.76	55
5	C6	B	20	36	1.80	72
6	C7	B	6	29	1.86	80
7	C7	A	15	32	1.83	70
8	C7	B	19	33	1.85	71
Mean ± SD	—	—	14 ± 5	34 ± 5	1.79 ± 0.06	67 ± 8
**PARA**						
1	T4	A	8	39	1.96	83
2	T4	A	24	41	1.76	72
3	T5	A	20	38	1.77	70
4	T8	A	7	24	1.85	64
5	T10	A	14	35	1.85	85
6	T10	A	19	32	1.68	58
7	T11	A	11	35	1.86	72
8	L3	A	18	34	1.86	72
Mean ± SD	—	—	15 ± 6	35 ± 5	1.82 ± 0.08	72 ± 9

A, motor- and sensory-complete; AIS, American Spinal Injury Association Impairment Scale; B, motor-complete and sensory-incomplete; C, cervical; L, lumbar; T, thoracic; TPI, time post-injury.

**Figure 2:**
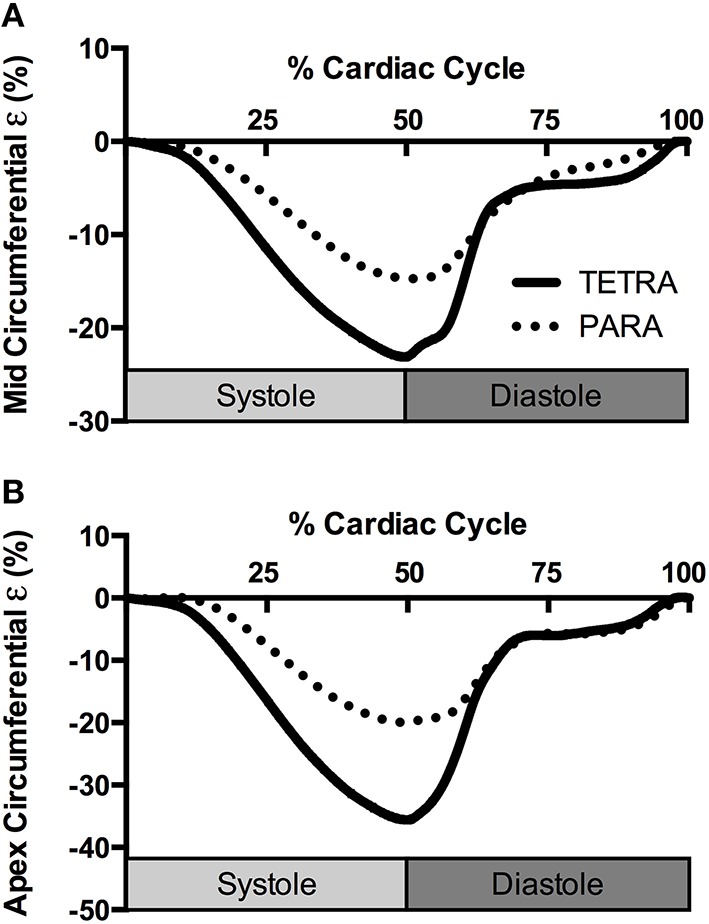
**Group averaged circumferential strain (ε) traces at the level of the papillary muscle (A) and apex (B) for TETRA (solid line) and PARA (dotted line), normalized to percentage of the cardiac cycle**.

Indices of LV structure and global function are presented in Table 2. Structure and global diastolic function was similar between groups, while global systolic function was reduced in TETRA compared to PARA including slower heart rates and smaller SV and $\dot{Q}$. Indices of LV mechanics are presented in Table 3. Basal Rot and RotR in both systole and diastole were higher in TETRA. The TETRA group also presented with higher circumferential ε (Figure 2) and diastolic SR at the mid and apical levels compared to PARA.

**Table 2:** **Indices of LV structure and global systolic and diastolic function**.

**Variable**	**TETRA**	**PARA**	***P*-value**	**Cohen's *d***
**STRUCTURE**				
Aortic annulus diameter (cm)	2.26 ± 0.13	2.38 ± 0.15	0.118	0.85
LVID d (cm)	4.86 ± 0.40	4.92 ± 0.21	0.341	0.19
SWT d (cm)	0.91 ± 0.06	0.90 ± 0.10	0.869	0.12
PWT d (cm)	0.84 ± 0.05	0.83 ± 0.12	0.956	0.11
LVID s (cm)	3.24 ± 0.22	3.32 ± 0.43	0.635	0.23
SWT s (cm)	1.27 ± 0.05	1.18 ± 0.16	0.177	0.76
PWT s (cm)	1.20 ± 0.10	1.26 ± 0.10	0.215	0.60
RWT	0.34 ± 0.04	0.34 ± 0.05	0.789	0.00
LVM Index (g·m^−2^)	79.3 ± 10.1	75.8 ± 9.4	0.491	0.36
EDV (ml)	106 ± 9	117 ± 10	**0.036**	1.16
ESV (ml)	44 ± 3	46 ± 11	0.551	0.25
**GLOBAL SYSTOLIC FUNCTION**				
SV (ml)	62 ± 9	71 ± 6	**0.016**	1.18
Heart rate (bpm)	57 ± 11	71 ± 6	**0.008**	1.58
$\dot{Q}$ (L/min)	3.5 ± 0.6	5.0 ± 0.9	**0.002**	1.96
EF (%)	59 ± 4	61 ± 7	0.394	0.35
**GLOBAL DIASTOLIC FUNCTION**				
E (cm/s)	86 ± 12	84 ± 7	0.733	0.20
A (cm/s)	43 ± 6	47 ± 12	0.373	0.42
E/A	2.02 ± 0.18	1.90 ± 0.50	0.544	0.32
IVRT (ms)	81 ± 6	75 ± 14	0.326	0.56

Data are mean ± SD. A, late transmitral filling velocity; d, end-diastolic; E, early transmitral filling velocity; EDV, end-diastolic volume; EF, ejection fraction; ESV, end-systolic volume; IVRT, isovolumetric relaxation time; LVID, left ventricular internal diameter; LVM, left ventricular mass; PWT, posterior wall thickness $\dot{Q}$, cardiac output; RWT, relative wall thickness; s, end-systolic; SV, stroke volume; SWT, septal wall thickness. Bolded values in P-value column indicate significant between-group differences.

**Table 3:** **Peak systolic and diastolic LV mechanics**.

**Variable**	**TETRA**	**PARA**	***P*-value**	**Cohen's *d***
**PEAK (SYSTOLE)**				
Basal Rot (degrees)	−7.3 ± 2.2	−4.4 ± 1.1	**0.005**	1.67
Apical Rot (degrees)	13.4 ± 3.3	14.4 ± 9.2	0.791	0.14
Twist (degrees)	19.5 ± 3.4	16.7 ± 10.3	0.527	0.37
ε_l_ (%)	−18 ± 3	−17 ± 2	0.344	0.39
ε_*r*_ (%)				
Basal Level	34 ± 11	30 ± 19	0.568	0.26
Mid Level	34 ± 16	32 ± 11	0.774	0.15
Apical Level	33 ± 17	13 ± 7	0.060	1.54
ε_*c*_ (%)				
Basal Level	−19 ± 7	−14 ± 5	0.147	0.82
Mid Level	−23 ± 4	−15 ± 6	**0.010**	1.57
Apical Level	−36 ± 10	−23 ± 5	**0.010**	1.64
Basal RotR (degrees·s^−1^)	−84 ± 26	−59 ± 21	**0.047**	1.06
Apical RotR (degrees·s^−1^)	91 ± 30	89 ± 28	0.874	0.07
Twist velocity (degrees·s^−1^)	129 ± 26	110 ± 31	0.218	0.66
SR (s^−1^)				
SR_l_	−0.92 ± 0.20	−0.91 ± 0.18	0.904	0.05
SR_r_ basal	1.90 ± 0.35	1.87 ± 1.0	0.946	0.04
SR_r_ mid	1.40 ± 0.61	1.69 ± 0.34	0.268	0.59
SR_r_ apical	1.71 ± 0.76	1.06 ± 0.51	0.173	1.00
SR_c_ basal	−1.25 ± 0.34	−0.93 ± 0.35	0.083	0.93
SR_c_ mid	−1.27 ± 0.17	−1.04 ± 0.38	0.137	0.78
SR_c_ apical	−2.40 ± 1.13	−1.85 ± 0.25	0.487	0.67
**PEAK (DIASTOLE)**				
Basal RotR (degrees·s^−1^)	55 ± 18	33 ± 12	**0.013**	1.44
Apical RotR (degrees·s^−1^)	−78 ± 34	−85 ± 42	0.751	0.18
Untwisting velocity (degrees·s^−1^)	−107 ± 22	−84 ± 30	0.110	0.87
SR (s^−1^)				
SR_l_	1.32 ± 0.32	1.15 ± 0.16	0.246	0.67
SR_r_ basal	−1.64 ± 0.56	−2.26 ± 1.38	0.273	0.59
SR_r_ mid	−2.00 ± 0.80	−2.44 ± 1.38	0.455	0.39
SR_r_ apical	−1.97 ± 1.19	−2.16 ± 1.61	0.840	0.13
SR_c_ basal	1.29 ± 0.32	1.31 ± 0.51	0.931	0.05
SR_c_ mid	1.90 ± 0.63	1.20 ± 0.51	**0.028**	1.22
SR_c_ apical	3.03 ± 0.71	1.99 ± 0.69	**0.009**	1.49

Data are mean ± SD. c, circumferential; ε, strain; l, longitudinal; r, radial; Rot, rotation; RotR, rotation rate; SR, strain rate. Bolded values in P-value column indicate significant between-group differences.

## Discussion

This is the first study to examine the influence of lesion level on resting LV structure, function and mechanics in Paralympic athletes with SCI. We observed no difference in LV dimensions or global diastolic function, while global systolic function was reduced in TETRA compared with PARA. Despite these observations, there were no indicators of LV mechanical dysfunction in the TETRA group, while PARA presented with attenuated systolic and diastolic mechanics. This observation may suggest a training induced improvement in mechanical efficiency in PARA that would increase their capacity to respond during an exercise bout.

The athletes included in this retrospective study were from two distinct sporting events. While we would consider any Paralympic athlete as highly trained, the nature of the training regiments for wheelchair rugby and alpine skiing are unique and incorporate different amounts of endurance and resistance exercise. While there is evidence in the able-bodied literature that endurance and resistance exercise training elicit differential cardiac adaptations (Spence et al., 2011), evidence from a cross-sectional examination of athletes with paraplegia found no differences in LV dimensions or global LV function between power-trained and endurance-trained athletes (Gates et al., 2002). Furthermore, endurance and resistance training in individuals with paraplegia has been shown to elicit similar improvements in peak oxygen uptake (Jacobs, 2009). Thus, it is unlikely that the differences observed between the TETRA and PARA groups are attributed to the differences in training modality. Both groups had similar weekly volumes of exercise training, and had been engaging in international competition for a similar duration. Additionally, the global LV indices observed in PARA athletes are comparable to values recently reported by De Rossi et al. (2014) in their examination of endurance trained athletes with paraplegia (i.e., wheelchair basketball, handball and tennis). While the training regiments for wheelchair rugby and alpine skiing are different, they do incorporate both aerobic and resistance exercises, which based on the literature to date, do not appear to elicit specific cardiac adaptations in individuals with SCI.

Exercise training may exert differential effects on global LV function depending on lesion level. De Rossi et al. (2014) demonstrated increased global diastolic function in athletes with tetraplegia and paraplegia relative to their sedentary peers with tetraplegia and paraplegia. However, SV was reduced in both sedentary and athletic groups with tetraplegia and only improved in athletes with paraplegia relative to sedentary individuals with paraplegia. Findings from the present study are in support of lesion level dependent differences in global function. We observed no difference in global diastolic function between TETRA and PARA, while SV and $\dot{Q}$ were lower in TETRA compared to PARA. The reduction in SV and subsequently $\dot{Q}$ are likely attributed to a smaller EDV given ESV was similar between groups, which implies reduced cardiac filling was likely responsible for the attenuated SV. LV dimensions were similar between groups; therefore the observed reduction in EDV is unlikely attributed to a smaller ventricle. While no investigations have directly compared LV dimensions between TETRA and PARA athletes, Kessler et al. (1986) observed smaller dimensions in sedentary individuals with tetraplegia compared to sedentary individuals with paraplegia. A more recent investigation using a pooled sample of athletes with tetraplegia and paraplegia observed no difference in LV dimensions when compared to able-bodied individuals and larger dimensions when compared to sedentary individuals with tetraplegia and paraplegia (De Rossi et al., 2014). Therefore, our observation of similar dimensions between the two groups is not unusual, and is likely attributed to the training status of our sample.

Contrary to the global systolic indices, systolic mechanics appear to be higher in TETRA relative to PARA. This includes higher basal Rot and circumferential ε at the level of the papillary muscle and apex, and a faster basal RotR. The ε-values reported in our TETRA sample are similar to values reported in the able-bodied literature (Yingchoncharoen et al., 2013). It is, therefore, unlikely that systolic mechanics are enhanced in the TETRA group, but rather the values in PARA group are lower. Head-down bed rest, which typically mirrors the cardiac adaptations following SCI, has been shown to elicit a reduction in SV, ε and systolic SR in able-bodied individuals (Kozakova et al., 2011; Scott et al., 2014). The observation of preserved systolic mechanics and reduced SV in TETRA was therefore unanticipated, and may be attributed to their hemodynamic state. In particular, reductions in afterload have been shown to increase systolic mechanics (Burns et al., 2010); therefore the lower blood pressure in our TETRA group may have created an environment where systolic mechanics can be maintained despite reductions in loading. Hypotension has consistently been demonstrated in sedentary (West et al., 2012b) and athletic (West et al., 2014) individuals with tetraplegia, and is attributed to the disruption of descending sympathetic input to the vasculature.

The observation of lower systolic and diastolic mechanics in our PARA group may be indicative of two scenarios: LV dysfunction/disease or a “resetting” of resting mechanics. Lower resting LV mechanics have been documented in clinical populations (Leung and Ng, 2010); however, we believe our finding is not pathological since global LV diastolic and systolic function (E/A and EF) were in a normal range (Lang et al., 2005; Dalen et al., 2010). The “resetting” of LV mechanics to lower resting values have been observed in high performance able-bodied athletes (Richand et al., 2007; Nottin et al., 2008), and while the mechanisms behind this adaptation are presently unknown, it is believed to create a large range for cardiovascular adjustments during exercise (Nottin et al., 2008). Both systolic and diastolic mechanics are increased during exercise in able-bodied individuals (Notomi et al., 2008; Stohr et al., 2011; Lee et al., 2012; Hensel et al., 2014). In addition to the obvious association between elevations in SV and ε, the augmentation of diastolic mechanics during exercise is key to promoting efficient diastolic filling, which in turn supports the elevations in SV (Notomi et al., 2008). It is presently unknown if exercise training in individuals with SCI elicits similar changes in LV mechanics as high-performance able-bodied athletes. Given cross-sectional evidence that athletes with paraplegia experience more favorable LV adaptations than athletes with tetraplegia (De Rossi et al., 2014), we postulate that involvement in high-performance sports and its associated large volume of exercise training may be capable of modifying LV mechanics to a lower resting value in PARA. Whether this modification has any impact on LV performance during exercise remains to be determined since LV performance *per se* has never been investigated during exercise post-SCI. Indeed, the few studies that have investigated the cardiac response to exercise in SCI have focused on measures of SV and report conflicting results, with some studies reporting increases in SV during arm-exercise (Davis et al., 1987; Hopman et al., 1992; Raymond et al., 1999), and others reporting no change (Davis and Shephard, 1988; Hopman et al., 1993, 1998; Raymond et al., 1999, 2001; Theisen et al., 2001). The discrepancy in these observations may be attributed to differences in the method used to estimate SV (ex. CO_2_ rebreathing, echocardiography, or impedance cardiography) and the acute exercise protocol (sub-maximal vs. maximal arm exercise). Nevertheless, the absence of apparent LV mechanical dysfunction at rest in high-performance athletes with SCI in the present study suggests any abnormal SV responses during exercise are likely attributable to reduced loading.

## Limitations

The absence of an able-bodied control group makes it difficult to draw conclusions regarding the direction of the LV mechanics. While global LV outcomes suggest the PARA group is free from LV dysfunction, it is possible that the attenuated LV mechanics values are indicative of impending cardiac dysfunction. Conversely, the TETRA group may have experienced an “enhancement” of LV mechanics to accommodate the cardiovascular requirements of their endurance sport. In order to fully understand the influence of lesion level of LV mechanics in Paralympic athletes, future research should attempt to evaluate athletes in similar sporting events, especially sports in which TETRA and PARA athletes are in competition against each other (ex. wheelchair basketball or Para-cycling team relays).

## Conclusion

In conclusion, high-performance athletes with TETRA and PARA experience differential changes in LV indices. While we would speculate these changes are attributed to lesion level, they may also be influenced by their training modality. TETRA athletes can be characterized as having normal diastolic function but reduced systolic function, an observation likely attributed to impaired venous return. On the other hand, PARA athletes demonstrated lower values for LV mechanics yet had no evidence of global systolic or diastolic dysfunction. We speculate this observation may be attributed to training induced improvements in LV efficiency rather than indicative of inherent LV dysfunction.

## Author contributions

KC—Conception of the work; data acquisition, analysis and interpretation; drafting and revising manuscript; approved final copy; and agrees to be accountable. CW—Conception of the work; data acquisition and interpretation; critically revised manuscript; approved final copy; and agrees to be accountable. AK—Conception of the work; data acquisition and interpretation; revising manuscript; approved final copy; and agrees to be accountable.

## Funding

Research at the Paralympic events was supported by donations from the International Collaboration on Repair Discoveries, Coloplast, Wellspect HealthCare, and the Shkreli Foundation. Dr. KC was supported by a Craig H. Neilsen Foundation Postdoctoral Fellowship (281863).

### Conflict of interest statement

The authors declare that the research was conducted in the absence of any commercial or financial relationships that could be construed as a potential conflict of interest.
